# The Cerebellum in Musicology: a Narrative Review

**DOI:** 10.1007/s12311-023-01594-6

**Published:** 2023-08-18

**Authors:** Stefan Evers

**Affiliations:** 1 Faculty of Medicine, University of Münster, Münster, Germany; 2 Department of Neurology, Krankenhaus Lindenbrunn, 31863 Coppenbrügge, Lindenbrunn 1 Germany

**Keywords:** Music, Cerebellum, Neuroimaging, Musicology

## Abstract

The cerebellum is involved in cognitive procressing including music perception and music production. This narrative review aims to summarize the current knowledge on the activation of the cerebellum by different musical stimuli, on the involvement of the cerebellum in cognitive loops underlying the analysis of music, and on the role of the cerebellum in the motor network underlying music production. A possible role of the cerebellum in therapeutic settings is also briefly discussed. In a second part, the cerebellum as object of musicology (i.e., in classical music, in contemporary music, cerebellar disorders of musicians) is described.

## Introduction

The cerebellum plays, besides its involvement in motor control, also a relevant role in cognitive processing including music processing. It is well known that executive functions, cognition, affect, and behavior are impaired by cerebellar damage; this syndrome is called “cerebellar cognitive affective syndrome (CCAS)” [1]. It has been shown by experimental trials and by functional studies on patients with cerebellar damage that CCAS can lead to deficits in emotion identification and regulation and in performing or perceiving music. In this chapter, a narrative review of the different links between music(ology) and the cerebellum is presented. In a first part, the cerebellum as object of musicology (i.e., in classicial music, in contemporary music, cerebellar disorders of musicians) is described. The second part is based on a previous review by the author which focused on cerebellar mechanisms in music perception and music performing [2].

## Musicology (Social Science) and the Cerebellum

The link between music and medicine sometimes also refers to the history of music, to the pathography of composers, and to other musicological aspects. With respect to the cerebellum, there is only very little known about this.

### Famous Musicians

The Russian composer Alfred Schnittke (1934 to 1998) suffered from a cerebellar bleeding in 1991. After this event, his compository extroverted polystylisme changed into a barer and withdrawn style [3].

Jacqueline Du Pres (1945–1987) was a British cellist, famous for her masterful interpretations and her passionate style of playing. In the age of 26, she slowly developed tremor and other cerebellar symptoms in addition to cerebral symptoms and was 2 years later diagnosed with multiple sclerosis [4]. She was unable to play the cello at that time due to hypaesthesia, probabaly stereoagnoisa and cerebellar symptoms such as tremor and ataxia.

The Finnish composer Jean Sibelius (1865–1957) suffered from an inherited tremor which increasingly lead finally to disability [5]. This tremor was one of his reasons to drink a lot of Whiskey and smoke cigars. It is likely that Sibelius’ tremor was just essential tremor; however, it could also have been a subtype of a spinocerebellar ataxia.

The French composer Maurice Ravel (1872–1937) suffered from a degenarive cerebral disorder which subtype is unknown [6]. He was described to have cerebellar symptoms such as ataxia and apraxia. However, the predominant symptoms were aphasia and amusia. Therefore, he could not have had a primarily cerebellar disorder, but the degeneration could have involved also the cerebellum.

Bedrich Smetana (1824–1884), a Czech composer, reported tremor, and ataxia from about 1874 onwards [7]. From the beginning of the 1880s, it became clear that Smetana was also psychotic. He was said to have paralytic dysarthria, unstable gait, and frequent falls. It is meanwhile proven by post-mortem autopsy that Smetana had acquired syphilis and that parts of his symptoms resulted from cerebellar involvement in progressive paralysis. Smetana was treated with mercury against his syphilis, and it might be that his symptoms were also a consequence of mercury poisoning. However, syphilis remains the primary cause of his symptoms since they started in part before onset of mercury treatment.

### The Cerebellum in Musical Compositions

**Fig. 1:**
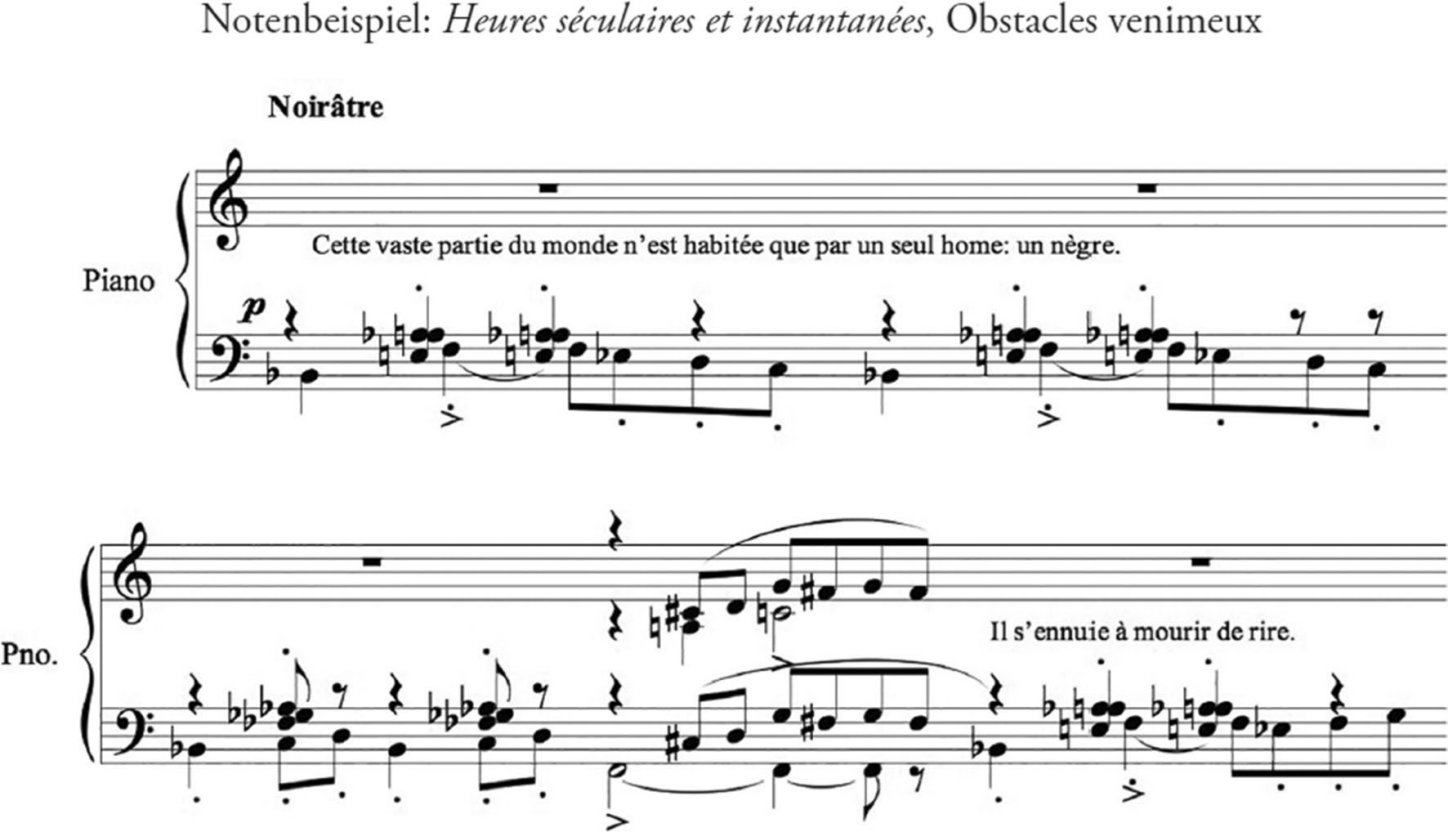
Cycle for piano “Heures séculaires et instantanées” (1914) from Erik Satie; first movement “Obstacles venimeux” (beginning)

The French composer Erik Satie (1866–1925) composed in 1914 the cycle for piano “Heures séculaires et instantanées”; in the first movement (“Obstacles venimeux,” see Fig. 1), the accompanying text to the music says “Cette vaste partie du monde n’est habitée que par un seul homme: un nègre. Il s’ennuie à mourir de rire. [...] Pour mieux penser, le nègre tient son cervelet de la main droite, les doigts de celle-ci écartes. De loin, il semble figurer un physiologiste distingué. Quatre serpents anonymes le captivent, suspendus aux basques de son uniforme que déforment le chagrin et la solitude réunis.” The English translation of the relevant text is “For better thinking, the negro holds his cerebellum with his right hand, the fingers of the latter spread apart.” This is a typical example for irony and sarcasm in the work of Erik Satie [8].

**Fig. 2:**
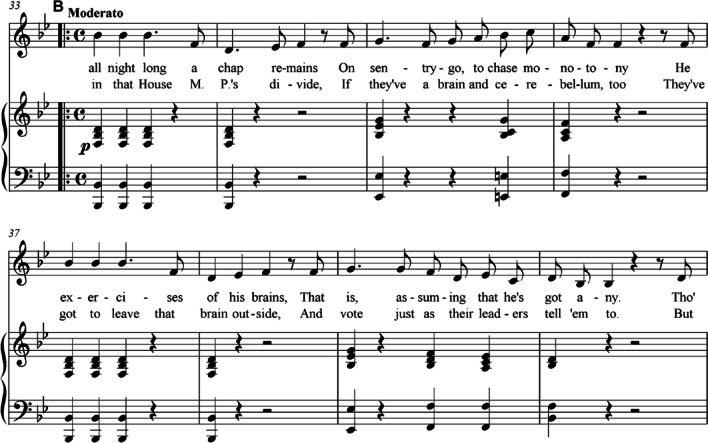
Act 2, No. 14 (excerpt) of the opera Iolanthe (1882) composed by Arthur Sullivan: a satiric description of the parliamentary system in the UK at that time

In the opera Iolanthe composed by Arthur Sullivan (1842–1900) in 1882, a satiric description of the parliamentary system in the UK at that time is given. In one aria (act II, no. 14, see Fig. 2), the person Private Willis sings:

“When in that House M.P.'s divide,

If they’ve a brain and cerebellum, too,

They’ve got to leave that brain outside,

And vote just as their leaders tell 'em to.”

In this text, the role of the cerebellum is just to raise the hand (or not) without thinking, this representing another satiric piece of music mentioning the cerebellum.

In the modern popular (mainly pop and rock) music, the term cerebellum can be found in several songs and was the name of one band and one album.

A US band from Louisville called Cerebellum existed in 1988/89. It recorded songs in the style of punk/hardcore. These songs were newly recorded in 2010 during a brief reunion and are available online. There is no obvious reason why the band called itself cerebellum. The Californian hardcore band Torena released an album in 2021 called cerebellum prison in which also a song has this title.

In the Internet databank on lyrics of rock, pop, and similar musical genres (http://www.lyrics.com, accessed March 1, 2023), 575 matches (out of 213,575 items in total; i.e., 0.003%) with the word cerebellum could be found, some of them are double because of featured songs etc. The use of the word cerebellum falls into different categories. Examples are given in Table 1:

- Sometimes the meaning/sense of the use of the word cerebellum does not disclose itself from the lyrics (example 1).
- It can occur in the context of love songs (example 2).
- It is used to structure a song (example 3, other parts of this example deal with brain, limbic system, and brain stem).
- In many songs, cerebellum is used to demonstrate (or substitute) balance (example 4; see also example 6). Also, in the context of rhythm, motor memory, and motor control, e.g., when dancing, the word cerebellum is used. This means a “correct” understanding of the cerebellum’s function.
- It is used just as an anatomical part of the brain, often in cruel songs (example 5).
- In the context of drug use, including the motor disturbances by drugs (example 6).

**Table 1:** Examples of mentioning the cerebellum in lyrics of modern pop or rock songs

Example 1: Jake Jessee, Tricaceron	
* Cerebellum mitochondria promise that you will call me back later*	
* Cerebellum mitochondria please pick up i really need a favor*	
Example 2: Masego, without title	
*Thinkin' I could make this many songs 'bout a lady*	
* When I haven't met her*	
* But she on me heavy, on my cerebellum*	
* If I say I love her, am I wrong?*	
Example 3: Young MC, Inside My Head	
Chorus 3: (spoken)	
* The cerebellum is located below the occipital lobe and behind the brain stem.*	
Example 4: Nassa, Cerebellum	
* I found the balance*	
* Cerebellum*	
* Kepler Kepler*	
* NASA NAsSA*	
Example 5: Cannibal corpse, Hatchet to the Head	
* Slit open crushed eyeballs dripping hanging from the sockets*	
* Steel cracked bone with fatal trauma to the cerebellum*	
* Useful lust of gore*	
Example 6: Green Day, Teenage Lobotomy	
* Slugs and snails are after me*	
* DDT keeps me happy*	
* Now I guess I'll have to tell 'em*	
* That I got no cerebellum*	
* Gonna get my Ph.D.*	
* I'm a teenage lobotomy*	
Example 7: Raizo, Poisonous Barz (feat. Graves)	
* Try and tell em what's your name*	
* Try and tie it to your cerebellum*	
Example 8: Sean Orlando, Real Recognize Real	
* I'm the mother fucking man*	
* Feel it in ya cerebellum nigga*	
Example 9: 46, Everything will make sense	
* Where do you get off and where do I go?*	
* Questions appear in my cerebellum*	
* Mind-numbing pain caused you to forgo*	
* Conclusions you thought of seldom*	
Example 10: Alex Vazco, Reign Supreme	
* Fire in my cerebellum,*	
* Hitting like Cerebella*	
* Develop skills to stay stellar*	

In many songs, the cerebellum is regarded as a part of the brain. As such, the word cerebellum is also used for cortical functions such as memory (example 7), emotions (example 8), and thinking (example 9). Sometimes, it is the impression that the word cerebellum was used just to create rhymes or special sound effects (example 9, example 10).

## Music Perception and the Cerebellum

Music perception involves a network of different brain structures depending on the type of music and the circumstances of perception. This typically also involves the cerebellum. Experimental brain research on music is difficult to perform because music is a complex cognitive task and its perception depends on biographical (educational and cultural) aspects. Thus, the different aspects of music processing have been examined as separate parameters and with different methods. This approach will be used in this narrative review to sumnmarize the findings on the link between the cerebellum and music perception.

### Rhythm and Timing

Musicians were asked to detect specific melodic, harmonic, or rhythmic errors. In this task, the cerebellum showed activation during all three exercises, but in the rhythm task cerebellar activation was twice as high as compared to the harmony and the melody task [9]. When comparing musicians and nonmusicians, nonmusicians show much stronger cerebellar activity in meter, tempo, and pattern discrimination whereas musicians show greater cerebellar activation during duration discrimination. This probably reflects differences in strategy, skill, and cognitive representation between musicians and nonmusicians.

Comparing patients with cerebellar atrophy, patients with focal cerebellar damage, and healthy control subjects in detecting rhythm frequency changes, only the cerebellar atrophy group presented significantly inferior results compared to control subjects [10]. This suggests that different cerebellar pathologies may differentially influence the ability to detect rhythm changes. Cerebellar damage seems also to affect the variability and stability of motor responses to rhythmic stimuli; and the involvement of the cerebellum in the memory for rhythm was verified by fMRI [11].

The cerebellum is also involved in the perception and synchronization with a musical beat as tested by the Harvard Beat Assessment Tests (H-BAT) [12]. There is a significant co-variation between performances on two perceptual tasks of the H-BAT associated with beat interval change discrimination (faster, slower) and gray matter volume variations in the cerebellum. The discrimination thresholds for the Beat Finding Interval Test (quarter note beat) were positively associated with gray matter volume variation in cerebellum lobule IX in the left hemisphere and [12]. The results demonstrate the importance of the cerebellum in beat interval discrimination skills suggesting that cerebellar gray matter and overall cerebellar integrity are important for rhythm discrimination abilities.

Distinct rhythmic elements (pattern, meter, tempo) engage different neural mechanisms. This was shown by recording brain activity of adult musicians and non-musicians with positron emission tomography (PET). For all rhythmic elements, focal activities in right or bilateral, areas of frontal, cingulate, parietal, prefrontal, temporal, and cerebellar cortices were detected [13]. In a clinical study, patients with cerebellar disorders showed significantly worse results in the metrum subtest in a test of musical ability [14] whereas the rhythm recognition was preserved.

Patients with cerebellar disorders are impaired in discriminating time intervals [15]. In addition, the cerebellum contributes to the production of a timed motor responses, in particular when it is complex and/or novel [16–18].

A meta-analysis on tasks related to music and timing revealed that tasks in these two domains recruit unique activations [19]. On the basis of the theory that the cerebellum can be seen as an internal timing device and evidence suggesting timing to involve knowledge of temporal order, activations for timing should represent a subset of activations for the rhythmic aspects of music processing. However, results in this meta-analysis revealed recruitment of distinct regions, with music-related tasks consistently demonstrating significant activation in right lobule IV/V, bilateral lobules VI and VIII, and timing uniquely activating the right lobule VI. The original publication [19] includes very detailed graphs on these cerebellar functions.

### Pitch and Timbre

Beside rhythm and its associated parameters, pitch and timbre are the other important basic parameters of music processing. The cerebellum is also involved in this processing. Patients with cerebellar degeneration, for example, were highly impaired in a pitch discrimination task and the degree of impairment correlated with the severity of their cerebellar ataxia [9]. In another study on patients with cerebellar degeneration, an ordinary pitch discrimination task was used [20]. The participants revealed strongly impaired discrimination abilities, and the amount of this deficit correlated with the degree of their cerebellar atrophy.

It is suggested that the supramarginal gyrus of the parietal lobe may function as a short-term pitch information storage site and that the cerebellum plays a critical role in performing pitch memory tasks [21]. Cognitive processes such as auditory information retention might depend on a mechanism separate from that of comparing two successive tones. In a PET study, cerebral blood flow changes pointed to a wider cortical and subcortical territory being involved when pitch retention was required versus the absence of memory load [22]. Summarizing the present literature, there is no doubt of a cerebellar involvement via multiple connections to cerebral cortical and subcortical areas in tasks requiring sensory data acquisition such as pitch.

The possible role of the cerebellum for sound processing was also tested in a pitch discrimination and a timbre discrimination task [23]. Participants performed a pitch and a timbre discrimination task prior and after receiving offline low-frequency transcranical magnetic stimulation (TMS) over their right cerebellum. Suppressing activity in the right cerebellum by inhibitory 1 Hz TMS worsened participants’ ability to discriminate pitch but not timbre. These findings point to a causal role of the cerebellum in at least certain aspects of sound processing and are important in a clinical perspective helping to understand the impact of cerebellar lesions on sensory functions.

Congenital amusia is a lifelong neurodevelopmental disorder of fine-grained pitch processing. The neural basis of congenital amusia has been investigated in an fMRI study with speakers of Cantonese, a tonal language [24]. Previous studies on non-tonal language speakers suggest that the neural deficits of congenital amusia lie in the music-selective neural circuitry in the right inferior frontal gyrus. However, it is unclear whether this finding can be generalized to congenital amusias in tonal languages. Cantonese-speaking amusic subjects exhibit abnormal brain activities in a widely distributed neural network during the processing of lexical tone and musical stimuli. While control subjects exhibit significant activation in the right superior temporal gyrus and in the cerebellum regardless of the lexical tone and music conditions, no activation was found in the amusic subjects in these regions including the cerebellum. This likely reflects a dysfunctional neural mechanism of relative pitch processing in the amusic subjects. No significant group difference was found in the right inferior frontal gyrus. These findings imply that the neural deficits in tonal language speakers might differ from those in non-tonal language speakers.

In a test of musical ability, patients with cerebellar disorders obtained significantly lower scores in the subtests for emotion recognition in music and for melody comparison than healthy control subjects [14]. This was more pronounced in patients with Machado-Joseph-Disease than in patients with cerebellar stroke.

In summary, there is evidence from activitation studies, from lesional studies, and from congenital amusia that the cerebellum is involved in pitch (but probably not in timbre) processing in a clinically relevant way.

### Music Identification

Identification and categorization of sounds as music is a higher-level cognitive skill which also involves the cerebellum. The most effective cue for music identification is a combination of pitch and duration, i.e., involving also cerebellar functions, at least for retrieving data from long-term memory for music [25]. Neuroimaging studies demonstrated distinct activated brain regions while participants accessed stored pieces of music [26–28]. It is suggested that when evaluating the familiarity of music, brain regions of retrieval from long-term memory as well as verbal and emotional processing would be included (i.e., bilateral anterior parts of the temporal lobe, superior temporal regions, and parahippocampal gyri) [28]. The process of recognizing familiar melodies proceeds in several steps. After sensory auditory information acquisition, a melody image is formed; then, melodies stored in long-term memory are retrieved and compared with this melody image.

How is the cerebellum involved in these pathways of music identification? In several PET series, a contralateral coactivation of auditory temporal cortex and lateral cerebellum suggests that they form a distributed circuit of auditory processing [9]. Based on neuroimaging [29] and anatomical [30] findings, the cerebro-cerebellar connectivity has been considered the basis for cerebellar involvement in music identification [31]. When listening passively, cerebellar activity has been detected in a study with healthy subjects. This activity even increased during pitch discrimination and was proportional to the difficulty of the task.

In a neuroimaging experiment [27], brain areas involved in the encoding and retrieval of melodies were investigated. Based on the presumption that once the first sequence of a familiar melody is played the successive part can be anticipated, the brain activation of healthy subjects during silent anticipation of familiar music was studied. There was a contrast between the activation during silent melody anticipation and the activation while listening to melodies. Besides activation of several cerebral areas, a significant bilateral activation of the posterior lateral cerebellum in the anticipation task could be observed. This shows that the cerebellum is even involved in music imagination.

The importance of a melody image for melody recognition was already described above. This image creation of music was found to imply a network of brain areas also involving the cerebellum [32]. Participants were displayed the titles and text of familiar lyrics during the actual process of melody imagery. This might have incited them to mental singing. Consequently, coactivation of motor and premotor areas (among others, activation was shown in an area containing the representation of tongue and lip movements in the cerebellum) were found during imagery, supporting the significance of an auditory motor loop and the importance of the cerebellum in terms of subvocalization during musical imagery.

Brain structures that are activated by emotional processing of short excerpts of film music successfully retrieved from episodic long-term memory comprise the bilateral superior temporal gyrus, right insula, right middle frontal gyrus, bilateral medial frontal gyrus, and the left anterior cerebellum [33]. Recognized (versus not recognized) old pieces of music showed a focused activation in the right inferior frontal gyrus and in the left cerebellum. In summary, specific brain networks related to memory retrieval and emotional processing of symphonic film music were identified.

In another study, the hypothesis was tested that listeners demonstrate different patterns of activation associated with music processing when encoding and retrieving culturally familiar and unfamiliar stimuli, with the latter evoking broader activation consistent with more complex memory tasks [34]. Using fMRI, US American and Turkish subjects listened to novel musical examples from their own culture and from an unfamiliar culture and identified which among a series of brief excerpts were taken from the longer examples. Both groups were more successful remembering music of their home culture. Greater activation for culturally unfamiliar music listening was found in the left cerebellar region, right angular gyrus, posterior precuneus, and right middle frontal area extending into the inferior frontal cortex. Subjects demonstrated greater activation in the cingulate gyrus and right lingual gyrus when engaged in recall of culturally unfamiliar music.

In six subjects with musical hallucinations following acquired deafness, PET was carried out to identify areas where brain activity increased as a function of the severity of the hallucination [35]. In a group analysis, no effect was demonstrated in the primary auditory cortices. Clusters of correlated activity were demonstrated in the posterior temporal lobes, the right basal ganglia, the cerebellum, and the inferior frontal cortices.

A cerebellar role in music-related information processing as well as in spatial-temporal tasks has been documented. Therefore, the cerebellar role in the association between spatial and musical domains was investigated by testing performances in embodied (EMR) or abstract (AMR) mental rotation tasks of subjects listening to Mozart’s Sonata KV 448, which is reported to improve spatial-temporal reasoning (a so-called Mozart effect), in the presence or in the absence of continuous theta burst stimulation (cTBS) of the left cerebellar hemisphere [36]. In the absence of cerebellar cTBS, music listening did not influence either an MR task, thus not revealing a “Mozart Effect.” Cerebellar cTBS applied before musical listening made subjects faster and less accurate in performing the EMR but not the AMR task. Thus, cerebellar inhibition by cTBS unmasked the effect of musical listening on motor imagery. These data support a coupling between music listening and sensory-motor integration in cerebellar networks for embodied representations.

In summary, the cerebellum plays also a role in music identifitcation including singing, in particular via pitch discrimination. This function of the cerebellum can be even detected in musical hallucinations. The major activation for music identification was found in the left anterior cerebellum.

### Emotion Processing

Besides various cerebral structures such as amygdala, hippocampus, and insula, the cerebellum is also involved in emotion processing [37] which is of course most important for music perception.

It was observed that children with treated benign (e.g., astrocytoma) or malignant (e.g., medulloblastoma) cerebellar tumors showed no significant differences in identifying a happy or sad emotion in music as compared to healthy control subjects [38]. Interestingly, the medulloblastoma subgroup revealed a poor performance in identifying sadness in music, pointing to a certain insensitivity to negative emotions. However, the study also suggests a restricted emotional cognitive control in both tumor groups. To date, the scientific literature suggests an impairment of patients with a cerebellar vermis lesion only with respect to anger or fear but not to other emotions [39].

The cerebellum participates in emotional responses to exteroceptive sensory stimuli [40]. Further, when investigating the brain regions involved in the identification of emotions (surprise/disgust/happiness/anger) induced by spoken words, a significant activation of the cerebellum was found [41]. Activation did also arise in parts of the frontal lobe indicating a functional relationship between these brain areas. In further neuroimaging studies, a neuronal network of brain regions including the cerebellum was detected in a task requiring emotional picture perception [42] and recognition of different emotions during focusing on the affective prosody of sentences with a neutral content [43].

However, the complete contribution of the cerebellum to the experience of emotions remains to be elucidated [39]. Cerebellar patients tend to overestimate the emotion happiness and to neglect the emotion anger. These two feelings can be categorized in pleasant and unpleasant emotion. With respect to this dichotomy, research has suggested that the neural systems contributing to these categories are tightly related [44]. Furthermore, a significantly increased cerebral blood flow of the left medial temporal lobe, of the occipito-temporal cortex, and of the cerebellum was found when viewing unpleasant pictures as compared to viewing neutral or pleasant pictures. Neuroimaging detected that cerebellar damage would impede to recruit brain structures which were normally attributed to the processing of unpleasant emotion [45]. Instead, an alternative neural circuitry is generated for this function, pointing to the evolutionary essential importance of fear experience preservation after cerebellar damage. This highlights the cerebellum as a structure of a dynamic network [45], concordant with brain circuitry in general. In this original publication [45], both structural and functional graphs explain the role of the cerebellum in emotional experience.

## Music Production and the Cerebellum

It is obvious that the cerebellum is an important part of the network regulating music production. Which specific mechanisms have been detected to date to better understand the role of the cerebellum in music production?

### Physiology

The cerebellar hemodynamic responses in highly skilled keyboard players and control subjects during complex tasks requiring unimanual and bimanual finger movements were investigated [46]. Both groups showed strong hemodynamic responses in the cerebellum during the task conditions with generally stronger hemodynamic responses in the cerebellum of the non-musicians. It was concluded that, due to long-term motor practice, a different cerebellar activation pattern can be visualized in keyboard players. For the same movements, fewer neurons need to be recruited. The different volume of the activated cortical areas might therefore reflect the different effort necessary for motor performance in both groups.

Several studies have shown that motor-skill training over extended time periods results in reorganization of neural networks and changes in brain morphology. Yet, little is known about such changes in the vocal system. Highly accomplished opera singers, conservatory level vocal students, and laymen were examined during singing of an Italian aria by neuroimaging techniques [47]. Training of vocal skills is accompanied by increased functional activation of bilateral primary somatosensory cortex representing articulators and larynx. At the subcortical level, expert singers showed increased activation in the basal ganglia, the thalamus, and the cerebellum. A regression analysis of functional activation with accumulated singing practice confirmed that vocal skills training correlates with increased activity of a cortical network together with increased involvement of implicit motor memory areas at the subcortical and cerebellar level.

Sung language and spoken language share many common features (physiology for articulation and perception as well as phonology, phonotactics, syntax, and semantics of the underlying language), although they differ in certain vocal and prosodic aspects. A review of the literature on perception and production of singing and speech [48] revealed considerable overlap in the lateral aspect of the VI lobule of the posterior cerebellum, a region known to somatotopically represent the lips and tongue. This region may instantiate internal models of vocal tract articulation that simulate well-learned phonological and/or segmental articulatory-auditory/orosensory mappings utilized for both speech and singing. A left cerebellar hemispheric specialization for processing of singing and right specialization for processing of speech is suggested, both in the VI lobule of the cerebellum, inferior to that found for representing both speech and singing. Given the crossed pattern of cerebellar-cortical anatomical connectivity, the findings are consistent with the hypothesis that the right cerebellum differentially processes high-pass filtered information (segmental properties) and the left cerebellum differentially processes low-pass filtered information (prosodic, melodic properties).

Activation of different brain regions from 13 professional cellists while interpreting Baroque and contemporary excerpts inside an MRI scanner was acquired [49]. Activation and connectivity brain maps showed common cortical motor and sensorial regions in both interpretation styles, but with different hemispheric intensity levels. However, certain auditory and motor regions were only activated during Baroque: the Heschl’s and superior frontal gyri, the planum temporale, and caudate. When playing contemporary music, the main origin of activation appeared in the cerebellar vermis, insular cortex, and parietal operculum. These discrepancies are probably attributed to different cognitive, sensory, and motor demands underlying the musical interpretation of each style.

The functional brain activation during singing and cello playing within the same individuals was compared to determine the extent to which arbitrary auditory-motor associations, like those required to play the cello, co-opt functional brain networks that evolved for singing. Musical instrument playing and singing both require highly specific associations between sounds and movements; therefore, it is often assumed that their neural underpinnings are highly similar. However, singing is an evolutionarily old human trait, and the auditory-motor associations used for singing are also used for speech and non-speech vocalizations. The pitch range of the cello is similar to that of the human voice, but cello playing is completely independent of the vocal apparatus, and can therefore be used to dissociate the auditory-vocal network from that of the auditory-motor network [50]. It was found that brain activity during cello playing directly overlaps with brain activity during singing in many areas within the auditory-vocal network. These include primary motor, dorsal pre-motor, and supplementary motor cortices, the primary and periprimary auditory cortices within the superior temporal gyrus including Heschl’s gyrus, anterior insula, anterior cingulate cortex, and intraparietal sulcus, and cerebellum but, notably, exclude the periaqueductal gray and basal ganglia.

### Cerebellum Morphological Plasticity

Numerous cross-sectional and observational longitudinal studies show associations between exercise/expertise and regional brain anatomy. This is also true for the cerebellum.

In a discordant monozygotic (identical) twin design, expertise-dependent effects on neuroanatomy using musical training as model behavior were studied, while essentially controlling for genetic factors and shared environment of upbringing [51]. Monozygotic twins who were highly discordant for piano practice showed that the musically active twin had greater cortical thickness in the auditory-motor network of the left hemisphere and more developed white matter microstructure in relevant tracts in both hemispheres and the corpus callosum. Furthermore, the volume of gray matter in the left cerebellar region (comprising lobules I–IV + V) was larger in the playing group. This finding supports the hypothesis that a significant portion of the differences in brain anatomy between experts and non-experts depends on causal effects of training.

Professional keyboard players were shown to have larger cerebellar volumes than matched non-musicians by analyzing high-resolution T1-weighted MR images from a large prospectively acquired database [52]. Significantly greater absolute and relative cerebellar volume but not total brain volume was found in male musicians compared to male non-musicians. Lifelong intensity of practice correlated with relative cerebellar volume in the male musician group. In the female group, there was no significant difference noted in volume measurements between musicians and non-musicians. It was proposed that the significantly greater cerebellar volume in male musicians and the positive correlation between relative cerebellar volume and lifelong intensity of practice represents structural adaptation to long-term motor and cognitive functional demands in the human cerebellum.

In another study, the relationships between regional cerebellar volumes, early musical training, and timing performance were investigated [53]. Adult musicians and non-musicians performed a standard finger tapping task, and cerebellar gray and white matter volumes were measured. Early-trained musicians had reduced volume in bilateral cerebellar white matter and right lobules IV, V, and VI, compared to late-trained musicians. Strikingly, better timing performance, greater musical experience, and an earlier age of start of musical training were associated with smaller cerebellar volumes. Better timing performance was specifically associated with smaller volumes of right lobule VI. Collectively, these findings support the sensitivity of the cerebellum to the age of initiation of musical training and suggest that lobule VI plays a role in timing. The smaller cerebellar volumes associated with musical training and timing performance might reflect a more efficiently implemented low-level timing and sensorimotor process.

Using drumming as a demanding multimodal motor training, cerebellar lobular volume and white matter microstructure, as well as cortical thickness of healthy non-musicians before and after learning to drum, were compared to age-matched control participants [54]. After 8 weeks of group drumming instruction, the cerebellum significantly changed its gray (volume increase of left VIIIa, relative decrease of VIIIb and vermis Crus I volume) and white matter microstructure in the inferior cerebellar peduncle. These plastic cerebellar changes were complemented by changes in cortical thickness (increase in left paracentral, right precuneus, and right but not left superior frontal thickness), suggesting an interplay of cerebellar learning with cortical structures enabled through cerebellar pathways.

A specific phenomenon is the so-called metaplasticity in the cerebellum. Musicians starting very early their training have smaller brain areas (including the cerebellum) due to the early optimization of the brain networks. This results in a scaffold for later developments [55]. This publication shows the involved cerebral and cerebellar parts in different graphs.

In summary, it has been shown that musical training induces morphological changes in the cerebellum independently from the type of musical performance.

## Therapeutic Implications

Music is commonly used to facilitate or support movement, and increasingly used in movement rehabilitation. Additionally, there is some evidence to suggest that music imagery, which is reported to lead to brain signatures similar to music perception, may also assist movement. One study revealed that moving to music compared with self-paced movement resulted in a significantly increased activation in the left cerebellum VI. Moving to imagined music led to significantly more activation in pre-supplementary motor area (pre-SMA) and right globus pallidus than to self-paced movement. When hearing music and music imagery were contrasted directly, movements in the music condition showed significantly more activity in left hemisphere cerebellum VII and right hemisphere and vermis of cerebellum IX, while music imagery condition revealed more significant activity in pre-SMA. These results suggest that cueing movement with actual or imagined music impacts upon engagement of motor network regions during movement. These results may have implications for the applicability of auditory cueing in movement rehabilitation [56].

Music can also be part of therapies for patients with Alzheimer’s disease with improvements in agitation, anxiety, and behavioral symptoms. After a training period with a personalized music listening program with Alzheimer’s patients, a specific activation of the supplementary motor area was shown, which has been associated with memory for familiar music that is typically spared in early Alzheimer disease. Also, widespread increases in functional connectivity in corticocortical and corticocerebellar networks following presentation of preferred musical stimuli were detected [57] showing the neurophysiological baseline for the role of music in the treatment of Alzehimer’s patients.

The interaction and the therapeutic relationship between the patient and the therapist in Music Therapy (MT) provides an additional element in need of investigation. In the course of a pilot study, these problems were approached and reduced to the simple observation of pattern alteration in the brains of four individuals with Unresponsive Wakefulness Syndrome (UWS) during MT. Each patient had three PET investigations: (i) during a resting state, (ii) during the first exposure to MT, and (iii) during the last exposure to MT. Two patients in the MT group received MT for 5 weeks between the 2nd and the 3rd PET (three times a week), while two other patients in the control group had no MT in between. Tracer uptake was measured in the frontal, hippocampal, and cerebellar region of the brain. With certain differences in these three observed brain areas, the tracer uptake in the MT group was higher (34%) than that in the control group after 5 weeks. The preliminary results suggest that MT activates the three brain regions described above even in patients with UWS [58].

## Perspectives

This narrative review shows that the cerebellum plays a role in music perception and production although only single aspects are known and many others are still unknown. It would be of interest to explore the role of the cerebellum in the global perception of music rather than in single aspects such as rhythm or pitch perception. Also, it would be of interest whether the cerebellum contributes to what we call nowadays musical ability. Cerebral damage seems to impact music perception and production much more than cerebellar damage, but does this mean that there is a hierarchy or is the cerebellum an indispensable part of a brain network in wich all parts are of the different importance? In total, much more studies have been performed on the cerebrum than on the cerebellum to investigate its importance for music perception.

Form the view of historical musicology, the cerebellum plays a very minor role in musical compositions and in the ability to compose. The majority of musical compositions in the history including modern music use the word cerebellum in a satiric manner and often in a wrong way (i.e., contributing properties to the cerebellum which are not part of its physiological role). This might result from the word itself which is in English a diminutive from cerebrum (as is in German “Kleinhirn” which means “little brain”). Maybe, with increasing knowledge of the role and function of the cerebellum in the general population, the use of the word cerebellum in cultural and social sciences will change in the future.

## Data Availability

Not applicable.
